# Electrical Properties of Aluminum Nitride Thick Films Magnetron Sputtered on Aluminum Substrates

**DOI:** 10.3390/ma15062090

**Published:** 2022-03-11

**Authors:** Daniele Desideri, Enrico Bernardo, Alain Jody Corso, Federico Moro, Maria Guglielmina Pelizzo

**Affiliations:** 1 Department of Industrial Engineering, University of Padova, via Gradenigo 6/a, 35131 Padova, Italy; federico.moro@unipd.it; 2 Department of Industrial Engineering, University of Padova, via F. Marzolo 9, 35131 Padova, Italy; enrico.bernardo@unipd.it; 3 Institute for Photonics and Nanotechnologies (CNR-IFN), National Research Council of Italy, via Trasea 7, 35131 Padova, Italy; alain.corso@pd.ifn.cnr.it; 4 Institute for Electronics, Information Engineering and Telecommunications (CNR-IEIIT), National Research Council of Italy, via Gradenigo 6/b, 35131 Padova, Italy; pelizzo@dei.unipd.it

**Keywords:** AC conductivity, *ε*_33_ permittivity, aluminum nitride, d_31_ piezoelectric coefficient, magnetron sputtering, thick film

## Abstract

The realization of a c-axis oriented aluminum nitride thick film on aluminum substrates is a promising step in the development of transducers for applications with a working temperature up to about 600 °C. The present paper deals with the realization of AlN thick films by means of reactive magnetron sputtering with a pulsed DC power supply, operating in continuous mode for 50 h. Two values (0.4 and 0.8) of nitrogen concentration were used; operative pressure and power were set at 0.3 Pa and 150 W, respectively. The thickness of the obtained aluminum nitride films on the aluminum substrate, assessed with a profilometer, varied from 20 to 30 µm. The preferential orientation of AlN crystals was verified by X-ray diffraction. Finally, as the main focus of the investigation, the films underwent electrical characterization by means of an LCR-meter used on a parallel plate capacitor set-up and a test system based on a cantilever beam configuration. AlN conductivity and *ε*_33_ permittivity were derived in the 100 Hz–300 kHz frequency range. Magnetron sputtering operation with nitrogen concentration equal to 0.4 resulted in the preferred operative condition, leading to a d_31_ piezoelectric coefficient, in magnitude, of 0.52 × 10^−12^ C/N.

## 1. Introduction

In situ process monitoring of materials working at high temperatures by means of piezoelectric transducers is an important issue in many applications of different fields, for example, aerospace or automotive [1,2]. Well known commercial piezoelectric materials consist of lead zirconate titanate (PZT) based ceramics, but their application is limited by their characteristic Curie temperature, not exceeding 350 °C [3].

Aluminum nitride (AlN) may offer a promising alternative for high-temperature applications, and AlN thin film sputtered on aluminum (Al) substrate is proposed for non-destructive testing applications with a working temperature up to about 600 °C [2]. The limitation of about 600 °C is due to the softening of the Al substrate at such a temperature; the AlN film actually remained well attached even after heating to 600 °C [2]. The good adhesion of AlN films on Al substrates, in our opinion, can be the basis for the development of transducers with a working temperature up to about 600 °C. With this in mind, with tests performed just at room temperature, the electrical properties of magnetron sputtered AlN thick films on Al substrates are here presented.

Magnetron sputtering is a well-known technique for growing AlN thin films oriented along the c-axis at low pressure [4,5]. The specific orientation of the AlN films allows us to obtain good values of the transverse piezoelectric coefficient. In this respect, one parameter in our investigation of the electrical characterization was the d_31_ coefficient. Regarding the magnetron sputtering operating conditions, the choice of the nitrogen to argon ratio is not immediate: with different substrates, the best results of AlN thin films oriented along the c-axis are reported by a few researchers at low nitrogen concentration [6,7] and by others at a high value of nitrogen concentration [8,9].

In order to perform the electrical characterization, one test system consisted of a simple parallel plate capacitor configuration made by coating AlN films deposited on Al substrates with silver conductive paint. This solution is very simple and allows us to estimate permittivity and conductivity with simple measurements of capacitance and resistance. A limit to this simple solution may be represented by the diffusion of the silver conductive paint into the AlN, especially if too low a value of the film thickness is used. To prevent this problem, we decided on the deposition of thick films.

A preliminary study of AlN films at 0.3 Pa and 0.5 Pa on Al substrates was described in [10]. The process length was equal to 20 h for each deposition, and the average value of film thickness was about 12 µm, corresponding to a deposition rate of about 10 nm/min. The best condition was achieved with a nitrogen concentration of 0.4 at 0.3 Pa.

For the research activity of this paper, the substrates set-up over the target was changed with respect to the configuration used for the preliminary study, but the target to substrate distance and the sputtering power remained the same. The research activity was performed at 0.3 Pa, and the process time length was extended to 50 h: films with a thickness of 20–30 µm were achieved. AlN thick films characterization was performed, and the obtained data were reported.

## 2. Materials and Methods

### 2.1. Magnetron Sputtering System

A magnetron sputtering system (bought from Kenosistec, Binasco MI, Italy) was used, with a stainless steel chamber (about 457 × 457 × 612 mm^3^), equipped with a large rectangular door (527 × 680 mm^2^). A single cathode was present, with a circular/planar (six inches diameter) aluminum 99.99% target. Argon and nitrogen gases were used in the magnetron sputtering operation.

#### Operative Conditions

Argon and nitrogen gas flows were set by a mass flow controller for each gas. The two gases were mixed with different values of flows, but the total value (i.e., the sum of nitrogen and argon flows) was kept at the same value (equal to 25 sccm) for all the operations here reported. By introducing the nitrogen concentration (N_2_ concentration) as the ratio between the nitrogen flow and the 25 sccm total flow, two values of this ratio were studied: 0.4 (i.e., 10 sccm for nitrogen and 15 sccm for argon) and 0.8 (i.e., 20 sccm for nitrogen and 5 sccm for argon). The operative pressure for the thick film deposition was set at 0.3 Pa. The operative pressure was obtained as a dynamic equilibrium condition by partial closure of a gate present between the sputtering chamber and the turbo vacuum pump.

The set values of pressure and nitrogen concentrations were chosen on the basis of the preliminary data reported in [10], where the best condition was achieved at 0.3 Pa with a nitrogen concentration of 0.4. At 0.3 Pa, another interesting condition was achieved with a nitrogen concentration of 0.8.

A pulsed DC power supply was used (TruPlasma DC 4001, Huettinger, Zielonka, Poland; 1 kW max output power). It operated with a repetition frequency of 50 kHz and a duty cycle of 90%. The output power value was set at 150 W.

A continuous system operation of 50 h was used for the deposition of each film.

The target-substrate distance for the operations reported in this paper was set at about 6 cm. The sputtering chamber base vacuum was always lower than 0.1 mPa. Presputtering for 10 min with argon gas, 0.3 Pa operative pressure and 25 sccm gas flow was performed in all the examined cases as a preliminary operation.

### 2.2. Substrates

For each operative condition, three substrates were prepared. Two substrates were parallelepiped aluminum pieces cut from a commercial AISI 1050A aluminum plate (bought from RS components), one bigger (substrate # 1, with dimensions about 116 mm × 12 mm × 2 mm) and a second one smaller (substrate # 2, with dimensions about 75 mm × 12 mm × 2 mm). A third substrate (substrate # 3, about 76 mm × 26 mm × 1 mm) was a commercial soda-lime glass.

For each operative condition, the following set-up was used. On substrate # 1, the other two substrates were fixed using adhesive Kapton tape placed on the back with respect to the surfaces facing the film growth. Substrate # 1 was place so it was leaning on the sustaining structure that is used for positioning the substrates over the target.

Substrate # 1 was exposed to the film growth for an area of about 110 mm × 12 mm. Regarding the other two substrates, a length of 35 mm from the top was exposed to film deposition in a central location over the target, the following 30 mm were masked by substrate # 1, and the remaining part (of 10 mm and 11 mm, respectively) was exposed to film deposition in a not central location over the target.

Substrate # 2, with the film deposition, was used for the measurement with the test systems described in Section 2.5. The films on substrates # 1 and # 3 were used for the measurements performed by X-ray diffractometer and profilometer.

#### Surface Smoothing of Aluminum Pieces

Preliminary smoothing of the surface exposed to film deposition of each aluminum piece (substrate # 1 and # 2) was performed by sequentially grinding the surface with three SiC abrasive papers (P600, P1000, and P1500); each one was driven manually for at least 10 min. After smoothing, the aluminum surface roughness was measured by a profilometer (indicated in Section 2.3) on a sample taken at random among the available pieces. Data were taken by three profilometric measurements (taken parallel to each other, and 50 µm apart one from the other) performed along a length of 1 mm. This resulted in surface roughness of 0.19 ± 0.04 µm (RMS value and variance). This result is comparable to the value of 0.14 ± 0.04 µm reported in the literature, obtained after polishing aluminum samples to a good surface finish [2].

### 2.3. Profilometer

The measurements of the AlN film thickness were performed by using a KLA-Tencor P-16+ stylus profilometer.

The thickness measurements were carried out on the step obtained by the masking arranged during the deposition process. The film’s height was obtained from the average of 10 profilometric measurements (taken parallel to each other and 50 µm apart one from the other) performed along a trace of 10 mm and using a stylus pressure force of 2 mg. Measurement averaging was performed after leveling each profilometric trace to remove the background and substrate bow and tilt.

### 2.4. X-ray Diffractometer

X-ray diffraction (XRD) analysis was performed using a Bruker AXS D8 Advance diffractometer in Bragg–Brentano configuration on coated glass sheets (Bruker, Karlsruhe, Germany—CuKα radiation, 1.5418 Å, 40 kV-40 mA, 2θ = 20–70°).

Phase identification was performed using the semi-automated Match!^®^ program package (Crystal Impact GbR, Bonn, Germany), supported by data from the PDF-2 database (ICDD—International Centre for Diffraction Data, Newtown Square, PA, USA).

### 2.5. Test Systems: Parallel Plate Capacitor and Cantilever Beam with Aluminum Nitride Layer

#### 2.5.1. Parallel Plate Capacitor

On substrate # 2, the AlN thick film was deposited on a length of 35 mm from the top and width equal to that of the Al substrate (12 mm). Silver conductive paint was applied over the AlN film, covering almost all the upper area of the film (about 33 × 12 mm). The aluminum substrate and the silver conductive area were the two electrodes of a parallel plate capacitor configuration, with the AlN thick film within the two electrodes. The electrode made of silver conductive paint was extended outside by means of a copper tape, with conductive glue, connected to the silver paint. Adhesive polyester tape and paper tape were used for the realization of electrical insulations. Finally, two wires were connected to the two electrodes, and the voltage between the electrodes was the output voltage that, versus frequency, was reported on an oscilloscope (the oscilloscope input impedance was 10 MΩ).

The permittivity and the conductivity were obtained using the relationships of the capacitance of a parallel plate capacitor and Ohm’s law:

*C*_*p*_ = *ε*_33_*A*/*h_p_*, (1)

*R_p_* = *A*/*σh*_*p*_, (2)

where *C_p_* (in F) is the capacitance of the parallel plane capacitor with area *A* (in m^2^) of the electrodes, *ε*_33_ (in F/m) is the AlN permittivity, *h_p_* (in m) is the thickness, *R_p_* (in Ω) is the resistance of the parallel plane capacitor configuration, and σ (in S/m) is the AlN conductivity. By setting *A* = 33 × 12 mm^2^, *h_p_* around 30 µm, and AlN permittivity *ε*_33_ around 80–90 pF/m [11], the capacitance values are ofthe order of 1 nF.

In this way, regarding permittivity, we assume that the imaginary part of permittivity is negligible and in agreement with that reported in the literature [12,13].

The *R_p_* and *C_p_* values in the frequency range 100 Hz–300 kHz were measured using an LCR meter (LCR-6300, Good Will Instrument Co., New Taipei City, Taiwan).

The contribution to the measurements due to the connection to the parallel plate capacitor (the two wires and the copper tape) were measured before the final assembling of the capacitive configuration. In the frequency range of analysis, the capacitance of the connections was around 20–30 pF, i.e., lower than 3% of the total parallel plate capacitor configuration. The resistance due to the connections was, in general, about 10–20 times higher than the measured resistance values of the total parallel plate capacitor configuration. With these data, the uncertainties due to the connections were considered acceptable and comparable with the uncertainties on the values of the film thickness and of the silver conductive paint area.

#### 2.5.2. Cantilever Beam with Aluminum Nitride Layer

A cantilever beam with a piezoceramic layer was realized in order to estimate the d_31_ transverse piezoelectric coefficient.

**Figure 1:**
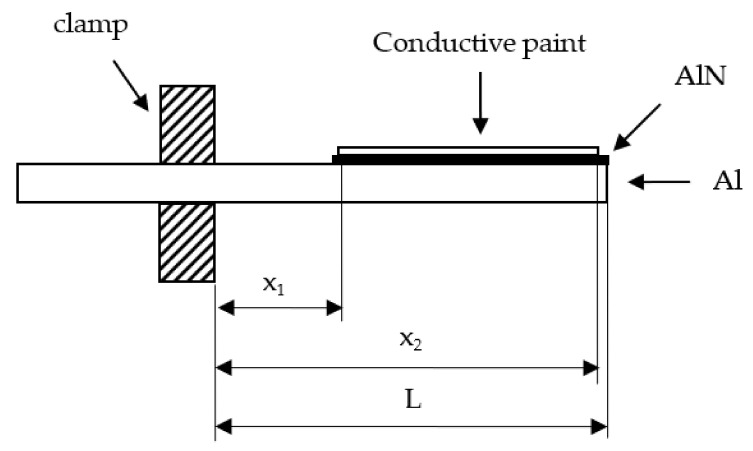
Schematic view (partial and out of scale) of the cantilever beam with piezoceramic layer test system.

The parallel plate capacitor set-up was mounted on an electrodynamic shaker (2007E of the Modal Shop Inc., Cincinnati, OH, USA) and clamped as a cantilever beam. The shaker was driven varying the frequency, and the acceleration imposed onto the system was measured by an accelerometer placed at the clamp of the cantilever beam. The acceleration rated value was 9.81 m/s^2^ (the gravity acceleration), RMS value, for all frequencies. In Figure 1, a partial schematic view (out of scale) of the cantilever beam is reported. Along the length of the beam, the distances from the clamp of the initial and final position of the silver conductive paint (*x*_1_ and *x*_2_, respectively) and the length L of the Al cantilever beam are indicated.

For the estimation of the magnitude of the AlN piezoelectric coefficient d_31_, the electromechanical model of a cantilevered piezoelectric energy harvester was applied [14]. The unimorph harvester model with harmonic excitation was used for the analysis of the voltage frequency response function, the ratio of the output voltage to the base acceleration. No base rotation was assumed and it was considered the first natural frequency (that, as seen experimentally for the analyzed cases, was around 500 Hz). With the value of the oscilloscope input impedance (*R_o_* = 10 MΩ) and the capacitance expected value of the parallel plate capacitor (*C_p_* around 1 nF), a time constant *τ_c_* = *R_o_C_p_* ≈ 10 ms was determined; therefore, with a first natural frequency of hundreds of hertz, the condition* ωτ_c_* >> 1 was satisfied. Under this condition, it resulted in [14]:

(3) $$\frac{v\left(t\right)}{-\omega^{2}Y_{0}e^{j\omega t}}=\frac{-m\varphi_{1}\gamma_{1}^{w}}{\chi_{1}\varphi_{1}+\omega_{1}^{2}-\omega^{2}+j2\zeta_{1}\omega_{1}\omega }\;,$$

where *v*(*t*) = *V*_0_ *e*^*jωt*^ is the output voltage [*V*], −*ω*^2^*Y*_0_ *e*^*jωt*^ is the base acceleration [m/s^2^], ω is the angular frequency [s^−1^], *ω*_1_ is the first natural angular frequency [s^−1^], *ζ*_1_ is the dimensionless damping ratio for the first mode, and m is the mass per unit length [kg/m] of the beam. For the unimorph energy harvester, with piezoelectric thickness *h_p_* [m] much smaller than the thickness of the substrate *h_s_* [m], it resulted in [14]:

φ_1_ = χ_1_/*C*_*p*._ (4)

The parameter *χ*_1_ [Cm^−1^kg^−1/2^] is given by:

(5) $$\chi_{1}=-d_{31}Y_{p}b\frac{h_{s}}{2}{\left.\frac{d\Phi_{1}\left(x\right)}{dx}\right|}_{x=x_{1}}^{x=x_{2}},$$

where *Y_p_* [N/m^2^] is the Young’s modulus of the piezoelectric material, *d*_31_ [C/N] is the piezoelectric constant, and *b* [m] is the width of the piezoelectric material (equal to the width of the aluminum substrate). The function *Φ*_1_(x) [kg^−1/2^] is [14]:

(6) $$\Phi_{1}\left(x\right)=\frac{1}{\sqrt{mL}}\left\{cosh\left(\frac{\lambda_{1}}{L}x\right)-cos\left(\frac{\lambda_{1}}{L}x\right)-\sigma_{1}\left[sinh\left(\frac{\lambda_{1}}{L}x\right)-sin\left(\frac{\lambda_{1}}{L}x\right)\right]\right\},$$

with *L* in *m*, and dimensionless parameters *λ*_1_ = 1.8751 and *σ*_1_ = 0.7341 [15].

With *C_p_* in F, *φ*_1_ is in CF^−1^m^−1^kg^−1/2^. The term $\gamma_{1}^{w}$ [m/kg^1/2^] is [14]:

(7) $$\gamma_{1}^{w}=\int_{x=0}^{x=L}\Phi_{1}\left(x\right)dx=\frac{2\sigma_{1}}{\lambda_{1}}\frac{L}{\sqrt{mL}}\approx 0.783\frac{L}{\sqrt{mL}},$$

By using Equations (3), (4), and (7), it was determined that the relationship between the RMS values of the output voltage (*V_RMS_*) [*V*] and the excitation acceleration (*E_RMS_*) [m/s^2^] is:

(8) $$V_{RMS}=E_{RMS}\frac{0.783\sqrt{mL}\frac{\left|\chi_{1}\right|}{C_{p}}}{\sqrt{{\left(\frac{\chi_{1}^{2}}{C_{p}}+\omega_{1}^{2}-\omega^{2}\right)}^{2}+{\left(2\zeta_{1}\omega_{1}\omega \right)}^{2}}},$$

An *m*_1_ term was added to Equation (8) to account for voltage offset in the experimental signal; therefore, data fitting was performed by the following function:

(9) $$V_{RMS}=m_{1}+\frac{m_{2}}{\sqrt{{\left(\omega^{2}-m_{3}^{2}\right)}^{2}+{\left(\omega m_{4}\right)}^{2}}}.$$

with *m*_1_ in *V*, *m*_2_ in V/s^2^, *m*_3_ in s^−1^, *m*_4_ in s^−1^.

From the *V_RMS_* experimental data and the data fit given by Equation (9), the *m*_2_ value was obtained. By using Equations (8) and (9), it resulted in:

(10) $$m_{2}=E_{RMS}0.783\sqrt{mL}\frac{\left|\chi_{1}\right|}{C_{p}}.$$

For all the cases reported in this paper, the following values were used: *x*_1_ = 16 mm, *x*_2_ = 49 mm, *L* = 50 mm, *b* = 12 mm, *h_s_* = 1.87 mm (measured value, corresponding to the nominal value of 2 mm of the aluminum pieces). The aluminum mass density value was taken to be 2700 Kg/m^3^, the aluminum nitride Young’s modulus was taken to be 345 GPa (at 298 K) [16]. Moreover, all the experimental data were obtained, setting *E_RMS_* = 9.81 m/s^2^. From Equations (5), (6), and (10), and using the given values, it was determined that the data fit of the output voltage *V_RMS_* (in Volt) gives *m*_2_, that is:

(11) $$m_{2}=k\frac{\left|d_{31}\right|}{C_{p}},\;k\approx 0.59\times 10^{9}\;Ns^{-2}.$$

The capacitance *C_p_* was measured at 500 Hz (i.e., near to the first natural frequency). Equation (11) shows that with the *m*_2_ data fit value and the measurement of the capacitance *C_p_*, the estimated value of the magnitude of *d*_31_ was determined.

## 3. Results

The adhesion of the films on substrate # 3 (the soda-lime glass) deposited with an N_2_ concentration equal to 0.4 was not good, and as was visually observed, a large detachment occurred. The situation regarding the N_2_ concentration equal to 0.8 was better.

The large detachment of the film from substrate # 3 for the case of 0.4 N_2_ concentration was confirmed, repeating the film deposition with the same operative conditions.

**Figure 2:**
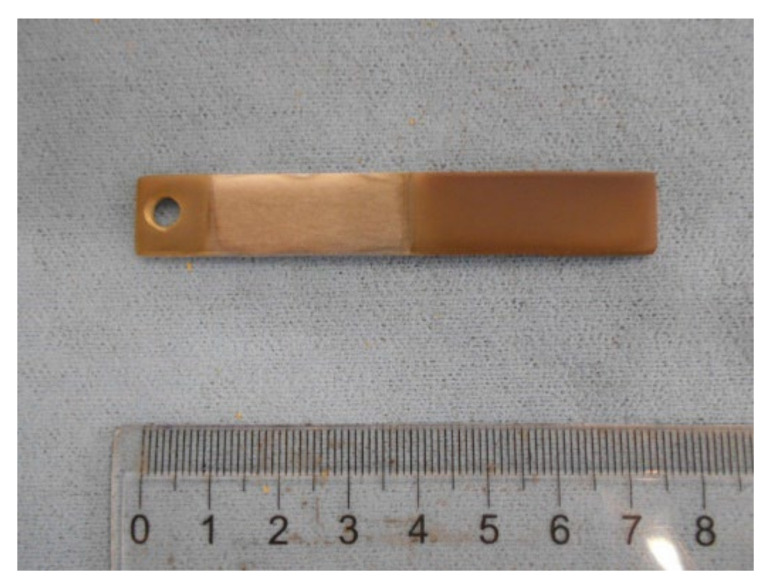
Substrate # 2 with AlN film magnetron sputtered. Moving from right to left: the film is deposited for a length of 35 mm; the following 30 mm were masked, and in the figure the aluminum substrate without film deposition is visible; finally, the film is on the last 10 mm (where a hole is present for connection).

The adhesion of the films on the aluminum substrates (# 1 and # 2) was visually observed and considered very good for both values of N_2_ concentration. In Figure 2, an example of the obtained AlN film on Al substrate is shown.

Therefore, X-ray diffractometer data and profilometer data relative to the film deposited on substrate # 3 were only reported for the operative condition of N_2_ concentration equal to 0.8.

Profilometer data relative to the film deposited on substrate # 1 were reported for both operative values (0.4 and 0.8) of N_2_ concentration.

### 3.1. Film Characterization

#### 3.1.1. Measurements of Film Thickness by Profilometer

The profilometer data relative to the AlN film deposited on substrate # 1 for the case of nitrogen concentration equal to 0.4 and 0.8 gave a film thickness value of 28.7 µm and 20.3 µm, respectively (reported in Table 1, with the corresponding values of the standard deviation).

The profilometer data relative to the AlN film deposited on substrate # 3 for the case of N_2_ concentration equal to 0.8 gave a film thickness value of 19.1 µm (reported in Table 1). This value is in good agreement with the value obtained for the film thickness deposited on substrate # 1.

For the following analyses, the values of film thickness obtained on substrate # 1 were used.

#### 3.1.2. Measurements of Capacitance versus Frequency

As reported, a parallel plate capacitor configuration was developed by applying a silver conductive paint over the AlN film deposited on the Al substrate.

The data for a sample realized with an N_2_ concentration of 0.4 and for a sample realized with an N_2_ concentration of 0.8 were obtained, and Equations (1) and (2) were used for the derivation of the permittivity *ε*_33_ and conductivity σ (as indicated, the thickness values measured on substrate # 1 were used, and *A* = 33 × 12 mm^2^ was taken).

**Figure 3:**
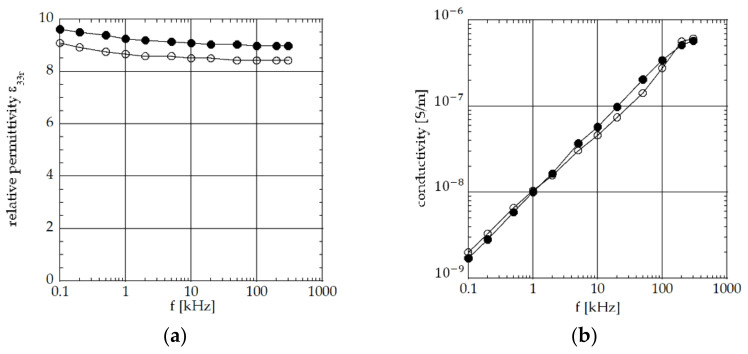
AlN thick films made at nitrogen concentrations of 0.4 (open circles) and 0.8 (full circles): (**a**) relative permittivity *ε*_33r_ versus frequency; (**b**) conductivity *σ* versus frequency.

In Figure 3a, the obtained relative permittivity *ε*_33r_ = *ε*_33_/*ε*_0_ (vacuum permittivity *ε*_0_ = 8.854 × 10^−12^ F/m) versus frequency is reported.

In Figure 3b, the obtained conductivity σ versus frequency is reported.

Regarding the relative permittivity, the two plots (nitrogen concentration of 0.4 and 0.8, respectively) are similar, with a limited difference. It can be noticed that this difference in percentage is comparable with the difference in percentage between the two measurements of AlN film thickness performed, respectively, on substrate # 1 and # 3 at an N_2_ concentration of 0.8 (reported in Table 1). The permittivity decreases as the frequency increases. Regarding the conductivity, the two curves (nitrogen concentration of 0.4 and 0.8, respectively) tend to overlap, and the value increases as the frequency increases.

#### 3.1.3. XRD Measurements

**Figure 4:**
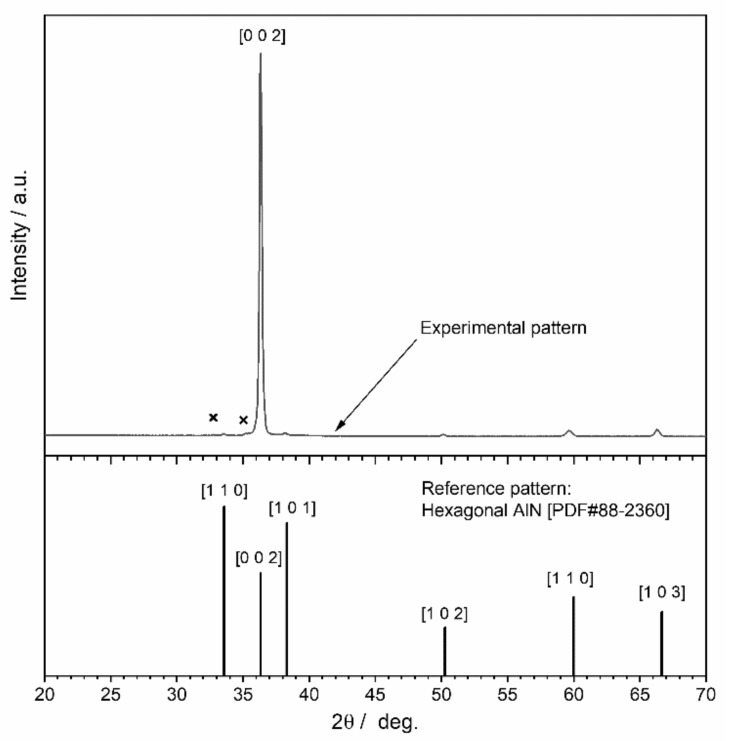
XRD data on substrate # 3 at nitrogen concentration of 0.8.

As indicated, a clear X-ray diffraction pattern could be obtained only for the film developed in the condition of nitrogen concentration of 0.8 (Figure 4). Most of the peaks in the pattern are consistent with the diffraction lines of hexagonal AlN (PDF#88-2360). Very weak extra peaks (marked with ‘X’ in Figure 4) are of doubtful attribution; a possible explanation, at least for the peaks at 2θ~35.5°, could come from traces of cubic AlN polymorph (PDF#80-0010). It must be noted that the experimental pattern matches with the reference pattern in terms of peak positions but not in terms of relative intensity. In fact, the very high intensity of the peak attributed to [0 0 2] planes could be due to anisotropic crystal growth along the c-axis; such preferential orientation nearly suppressed signals corresponding to other planes. In particular, peaks attributable to [1 1 0] and [1 0 1] planes, i.e., the strongest lines in the reference pattern, are hardly visible.

#### 3.1.4. Estimation of Transverse Piezoelectric Coefficient by Cantilever Beam Set-Up

**Figure 5:**
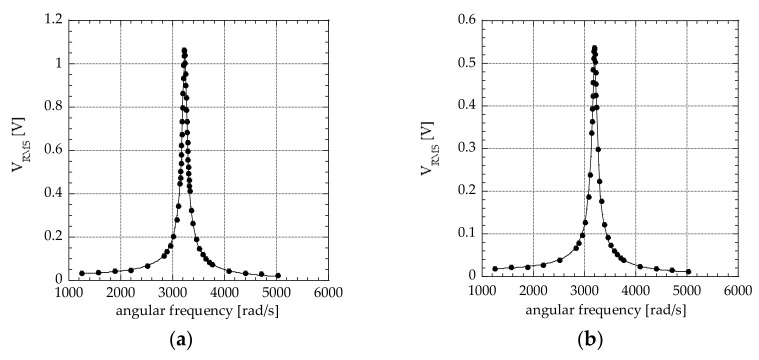
Output voltage (RMS value) V_RMS_ experimental data obtained by cantilever beam set up with E_RMS_ = 9.81 m/s^2^: (**a**) nitrogen concentration of 0.4; (**b**) nitrogen concentration of 0.8.

In Figure 5a,b, the data obtained with a thick film made at nitrogen concentrations of 0.4 and 0.8, respectively, are shown. A factor of about 2 is present in the output voltage between the two cases. The numerical fit performed with Equation (9) on the experimental data reported in Figure 5a,b gave the m_2_ parameter the values of 2.88 × 10^5^ V/s^2^ and 1.53 × 10^5^ V/s^2^ at nitrogen concentrations of 0.4 and 0.8, respectively. With capacitance values at 500 Hz of 1.07 nF and 1.62 nF, respectively, at 0.4 and 0.8 nitrogen concentration, by using Equation (11), it resulted in a magnitude of d_31_ of 0.52 × 10^−12^ C/N and 0.42 × 10^−12^ C/N, respectively, as reported in Table 2. The value of 0.52 × 10^−12^ C/N, even if it is not so high, is comparable with the experimental data obtained on AlN thin films reported in the literature [17].

## 4. Discussion

In the literature, both nitrogen concentrations (0.4 and 0.8) are considered appropriate for the realization of the AlN film [5]. In general, with increasing nitrogen concentration, typically, three operation modes of magnetron sputtering can be identified according to the coverage of the aluminum target by aluminum nitride. At low nitrogen concentrations, when the coverage is limited, a “metal mode” is identified; secondly, a “transition mode” is activated at higher concentrations up to a limit at which a “Poison mode” is established.

At low nitrogen concentrations, the deposition rate is higher since argon particles are heavier than nitrogen particles and are able to extract a higher number of aluminum atoms from the target. In agreement with this mechanism, our results show that the film thickness at a nitrogen concentration of 0.4 is greater than the thickness achieved at a nitrogen concentration of 0.8.

From the point of view of the realization of highly c-axis AlN films, some authors [7] suggest the adoption of a low nitrogen concentration. They claim that in this condition, more aluminum atoms are ejected from the target at high kinetic energies, and the impact of these energetic atoms on the growing film is such that the crystals with a close-packed (002) plane parallel to the substrate surface are more able to sustain such energies compared to other crystals with loosely packed planes; therefore, this results in the high c-axis orientation of the AlN films. On the contrary, some authors [8,9] report high nitrogen concentration as preferable, since more Al atoms sputtered from the target are able to react with N_2_ before reaching the substrate. When the substrate is reached, the newly formed AlN can orient itself with the other c-axis oriented grains.

Our results agree with the adoption of a low nitrogen concentration as the best condition for AlN films oriented along the c-axis: the highest value of transverse piezoelectric coefficient d_31_, clearly reflecting the growth of c-axis oriented film, was effectively achieved with a nitrogen concentration of 0.4.

Permittivity and conductivity of the AlN thick films magnetron sputtered on aluminum substrates were estimated in the frequency range 100 Hz–300 kHz. These results are in good agreement with the values reported in the literature at room temperature [12,13]. In particular, the conductivity data reported in Figure 3 show a type of behavior that is often described in the literature by the expression *σ* = *Aω^s^*, where A is a constant and s is an index that depends on temperature and frequency. From the data of Figure 3, it was determined that index s was 0.71–0.75, in agreement with the values reported in the literature [13].

## 5. Conclusions

Thick films of AlN on an aluminum substrate with visually good film adhesion were obtained.

The electrical characterization of the thick films, performed in the frequency range 100 Hz–300 kHz, confirmed the expected values for highly oriented AlN crystals. With a nitrogen concentration of 0.4, the obtained value for the piezoelectric coefficient d_31_ (0.52 × 10^−12^ C/N in magnitude) is reasonable, with a film thickness of about 30 µm.

Currently, the main limitation for the development of a transducer with a working temperature up to about 600 °C is that apart from the Al substrate and the AlN film deposited on it, other parts are not yet able to work up to 600 °C: the next step will be to improve the configuration in order to complete the set-up for operations up to 600 °C.

## Figures and Tables

**Table 1:** Thickness values (with corresponding values of standard deviation) of AlN films deposited on substrate # 1 and # 3 obtained by profilometer measurements. The films were deposited with magnetron sputtering at 0.3 Pa operative pressure and 150 W power value and nitrogen concentration set at 0.4 and 0.8, respectively.

N_2_ Concentration	Film Thickness on Substrate # 1 (µm)	Film Thickness on Substrate # 3 (µm)
0.4	28.7 ± 0.6	n.a.
0.8	20.3 ± 0.5	19.1 ± 0.5

**Table 2:** Magnitude of piezoelectric coefficient d_31_ derived from fit of data obtained by cantilever beam set-up with AlN films. AlN films were deposited on aluminum substrates by magnetron sputtering at 0.3 Pa operative pressure and 150 W power value and nitrogen concentration set at 0.4 and 0.8, respectively.

N_2_ Concentration	Magnitude of d_31_ (× 10^−12^ C/N)
0.4	0.52
0.8	0.42

## Data Availability

Data sharing is not applicable to this article.
